# Assessment of the Cytotoxic Effect of a Series of 1,4-Dihydropyridine Derivatives Against Human Cancer Cells

**Published:** 2016

**Authors:** Nima Razzaghi-Asl, Ramin Miri, Omidreza Firuzi

**Affiliations:** a *Department of Medicinal Chemistry, School of Pharmacy, Ardabil University of Medical Sciences, Ardabil, Iran. *; b *Drugs and Advanced Sciences Research Center, School of Pharmacy, Ardabil University of Medical Sciences, Ardabil, Iran.*; c *Medicinal and Natural Products Chemistry Research Center, Shiraz University of Medical Sciences, Shiraz, Iran.*

**Keywords:** Synthesis, Dihydropyridine, Anticancer activity, MTT assay

## Abstract

Cancer is a leading cause of death worldwide. Despite the availability of several chemotherapeutic drugs, there is still a great need for more efficient agents for a better management of cancer. In this contribution, a series of 11 1,4-dihydropyridines (1,4-DHPs) (4a, 4b and 7a-i) were synthesized and evaluated for their cytotoxic effect against MCF-7, LS180 and MOLT-4 cancer cell lines using MTT assay. Synthesized 2,6-dimethyl-3,5-bis-*N*-(aryl/heteroaryl) carbamoyl-4-aryl-1,4-dihydropyridines exhibited different potencies ranging from weak to good cytotoxic activities, while no activity could be recorded for 1,4-bis(2,6-dimethyl-3,5-dialkyloxylcarbonyl,4-dihydropyridine-4-yl) benzene compounds (4a and 4b). Tested DHP derivatives were more potent against MOLT-4 cells, when compared to LS180 and MCF-7 cells. Compounds 7d (IC_50 _= 28.5 ± 3.5 µM), 7a (IC_50 _= 29.7 ± 4.7 µM) and 7a (IC_50 _= 17.4 ± 2.0 µM) were the most potent derivatives against MCF-7, LS180 and MOLT-4 cells, respectively. It appeared that the introduction of *N*-thiazolyl carbamoyl group at the C3 and C5 positions of DHP ring enhanced the cytotoxic potential of these derivatives (compounds 7a-e). The findings of this study suggest that some of the thiazole substituted 1,4-DHPs may be candidates for further modifications towards the discovery of potent anticancer agents.

## Introduction

Cancer is characterized by uncontrolled cell growth and division. Nowadays cancer is an important cause of mortality worldwide that affects a huge number of people during their life (1). On the basis of recent reports, approximately 17 million cancer deaths per year might occur by 2030 (2). Despite the remarkable progress in the prevention and treatment of cancer, chemotherapy methods are hindered with significant limitations (3). Therefore development of efficient and selective antitumor agents is one of the fundamental challenges of researchers working in the field of pharmaceutical sciences. 

The 1,4-dihydropyridines (DHPs) are an important class of calcium channel blockers (4) and a common feature of various medicinal scaffolds such as anti-hypertensive (5), antidiabetic (6), antituberculosis (7), anti-coagulant (8), antidyslipidemic (9), antioxidant (9, 10), and multidrug resistance (MDR) reversal (11, 12) agents. 

**Scheme 1:**
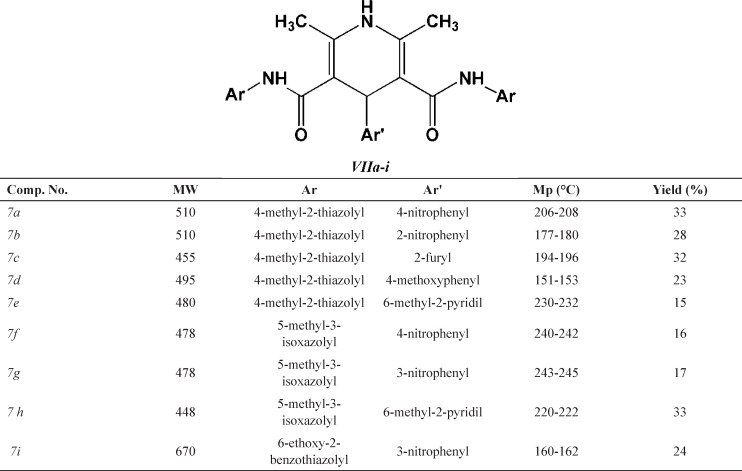
Chemical structure of dexniguldipine

Importantly, DHPs and other calcium channel blockers have demonstrated efficacy as anticancer agents in several studies (13-15). In this regard, several reports indicating anti-tumor activity of symmetric 3,5-dicarboxamide (16), 3,5-diketo (13, 17), 3,5-dicarboxylate (17, 18) and 3,5-dicyano (19) derivatives of 1,4-DHPs have been documented in the literature. Dexniguldipine (Scheme 1) is a prototype of the asymmetrical 1,4-DHPs exhibiting therapeutic activity in canine osteosarcoma (20).

Moreover, there are some reports highlighting the effect of DHPs on potentiation of anti-tumor activity of common chemotherapeutic drugs. For instance, structural conjugation of homocamptothecins with DHP scaffold enhanced the inhibition of DNA topoisomerase I (21). It has been also shown that 3,5-dibenzoyl-4-(2-methylthiazol-4-yl)-1,4-dihydro-2,6-dimethylpyridine and 3,5-diacetyl-4-[2-(2-chlorophenyl)thiazol-4-yl)]-1,4-dihydro-2,6-dimethylpyridine exhibited lower cytotoxic effect compared to doxorubicin, however, it could significantly potentiate doxorubicin cytotoxicity in T47D and RS cancer cells (13).

Considering the available evidence on the potential antitumoral effect of DHP structures and also based on our interest in assessment of biological activities of 1,4-DHP structures (22-24), herein we report the cytotoxic activities of new 2,6-dimethyl-3,5-bis-*N*-(aryl/heteroaryl) carbamoyl-4-aryl-1,4-dihydropyridine and 1,4-bis(2,6-dimethyl-3,5-dialkyloxylcarbonyl-1,4- dihydropyridine-4-yl) benzene derivatives on three different human cancer cell lines. 

## Experimental

*Materials and methods*

*Chemistry*

All the synthesized compounds were characterized by mass spectroscopy, IR and ^1^H NMR. IR spectra were recorded on a Nicolet FT-IR Magna 550 spectrophotometer. ^1^H NMR spectra were determined by a Bruker FT-500 MHz spectrometer in chloroform-d_1_ or DMSO-d_6_. All the chemical shifts were reported as δ values (ppm) against tetramethylsilane as an internal standard. The MS spectra were recorded using a Finnigan TSQ-70 spectrometer at 70 eV. CHN/CHNS analysis was performed using CHNS-932 Leco analyzer and the results were within ± 0.4% of the theoretical values.

*General procedure for the synthesis of 1,4-bis(2,6-dimethyl-3,5-dialkyloxycarbonyl-1,4-dihydropyridine-4-yl) benzenes (4a and 4b)*

A mixture of ammonium acetate 1 (3 mmol), corresponding alkyl-3-oxo butanoate 2 (4 mmol) and terephthalaldehyde 3 (1 mmol) was refluxed in 20 mL *iso*-propyl alcohol in an oil bath for 24 h. 

The progress of the reaction was monitored by TLC. On completion of the reaction, the solvent was removed to some extent under reduced pressure. The product was filtered, washed with small portions of cold methanol and then dried to afford the final product 4b. As for compound 4a, similar procedure was carried out while further purification by column chromatography and preparative TLC using ethylacetate/petroleum ether as eluent afforded the pure product. Physical characteristics data of the final compounds are summarized in Table 1.

*1,4-bis(2,6-dimethyl-3,5-diisobutyloxylcarbonyl-1,4-dihydropyridine-4-yl) benzene (4a)*

Pale yellow solid in 28%; m.p.: 232-234 °C;^ 1^H NMR (CDCl_3_) δ (ppm): 7.09-7.28 (d, 4H, CH-aromatic), 5.59 (brs, 2H, NH-DHP), 4.99 (s, 2H, C4-H DHP), 3.81 (d, 8H, CH_2_), 2.35 (m, 4H, CH), 1.90 (s, 12H, CH_3_-DHP), 0.88 (d, 24H, CH_3_); IR (KBr) *ν*_max_ (cm^-1^): 3343.5 (NH, DHP), 3098.8 (CH, aromatic), 2961.6 and 2874.6 (CH, aliphatic), 1698.2 (C=O, ester); MS m/z (%): 692 (M^+^, 36), 619 (34), 591 (72), 384 (100), 308 (100), 252 (21), 196 (50), 150 (20), 41 (6); Anal. Calcd. for C_40_H_56_N_2_O_8_: C, 69.34; H, 8.15; N, 4.04; Found: C, 69.45; H, 8.11; N, 4.18. 

*1,4-bis(2,6-dimethyl-3,5-diallyloxylcarbonyl-1,4-dihydropyridine-4-yl) benzene (4b)*

Pale yellow solid in 44%; m.p.: 208-211 °C;^ 1^H NMR (CDCl_3_) δ (ppm): 7.09 (d, 4H, CH-aromatic), 5.82-5.89 (m, 4H, CH-alkene), 5.59 (brs, 2H, NH-DHP), 5.11-5.16 (d, 8H, CH_2_-alkene), 5.02 (s, 2H, C4-H DHP), 4.55 (m, 8H, CH_2_), 2.33 (s, 12H, CH_3_-DHP); IR (KBr) *ν*_max_ (cm^-1^): 3306.7 (NH, DHP), 3081.4 (CH, aromatic), 2923.9 and 2853.1 (CH, aliphatic), 1717.8 (C=O, ester); MS m/z (%): 628 (M^+^, 36), 587 (34), 543 (72), 276 (100), 236 (88), 218 (81), 191 (37), 150 (20), 41 (35); Anal. Calcd. for C_36_H_40_N_2_O_8_: C, 68.77; H, 6.41; N, 4.46; Found: C, 68.95; H, 6.31; N, 6.60. 

*General procedure for the synthesis of N-aryl-3-oxo butanamides*

Required* N*-(aryl)-3-oxobutanamides were synthesized according to the modified method of Clemens via condensation of 2,2,6-trimethyl-1,3-dioxine-4-one with the appropriate arylamines (25). Detailed synthetic procedure to *N*-aryl-3-oxo butanamides may be found in our previous report (26).

*General procedure for the synthesis of 2,6-dimethyl-3,5-bis-N-(aryl/heteroaryl) carbamoyl-4-aryl-1,4 dihydropyridines (7a-i)*

Desired compounds (7a-i) were obtained via reaction of ammonium acetate 1 (2 mmol), corresponding *N*-arylacetoacetamides (5a-c) (2 mmol) and appropriate aldehyde (6a-d) (1 mmol) in 20 mL absolute ethanol in an oil bath within 24–48 h. Detailed synthetic procedure to compounds 7a-i may be found in our previous publication (26). Some characteristics of final products (7a-i) are summarized in Table 2. 

*Biological activity*

*Reagents and Chemicals*

RPMI 1640, fetal bovine serum (FBS), trypsin and phosphate buffered saline (PBS) were purchased from Biosera (Ringmer, UK).3-(4,5-Dimethylthiazol-2-yl)-2,5 diphenyltetrazolium bromide (MTT) was obtained from Sigma (Saint Louis, MO, USA) and penicillin/streptomycin was purchased from Invitrogen (San Diego, CA, USA). Cisplatin and dimethyl sulphoxide were obtained from EBEWE Pharma (Unterach, Austria) and Merck (Darmstadt, Germany), respectively.

*Cell lines*

MCF-7 (human breast adenocarcinoma), LS180 (human colon adenocarcinoma), and MOLT-4 (human acute lymphoblastic leukemia) cells were obtained from the National Cell Bank of Iran, Pasteur Institute, Tehran, Iran. All cell lines were maintained in RPMI 1640 supplemented with 10% FBS, and 100 units/mL penicillin-G and 100 m g/mL streptomycin. Cells were grown in monolayer (MCF-7 and LS180) or in suspension (MOLT-4) cultures.

*Cytotoxic effect*

Cell viability following exposure to synthetic compounds was estimated by using the MTT reduction assay (22, 27). LS180 and MOLT-4 cells were plated in 96-well microplates at a density of 5 × 10^4^ cells/mL (100 μL per well), while MCF-7 cells were plated at a density of 3 × 10^4 ^cells/mL. Control wells contained no drugs and blank wells contained only growth medium for background correction. After overnight incubation at 37 °C, half of the growth medium was removed and 50 μL of medium supplemented with 10, 25, 50 and 100 μM of synthetic compounds were added. Compounds were all first dissolved in DMSO and then diluted in medium so that the maximum concentration of DMSO in the wells did not exceed 0.5%. Cells were further incubated for 72 h. At the end of the incubation time, the medium was removed and MTT was added to each well at a final concentration of 0.5 mg/mL and plates were incubated for another 4 h at 37 °C. Then formazan crystals were solubilized in 200 μL DMSO. The optical density was measured at 570 nm with background correction at 655 nm using a Bio-Rad microplate reader (Model 680). The percentage of inhibition of viability compared to control wells was calculated for each concentration of the compound and IC_50_ values were calculated with the software CurveExpert version 1.34 for Windows. The absorbance of wells containing no cells was subtracted from sample well absorbance before calculating the percentage of inhibition. Each experiment was repeated 4 times. 

**Table 1:** Chemical structures of the aromatized 1,4-bis(2,6-dimethyl-3,5-dimethoxylcarbonyl-1,4-dihydropyridine-4-yl) benzene derivatives (*IVa* and *IVb*)

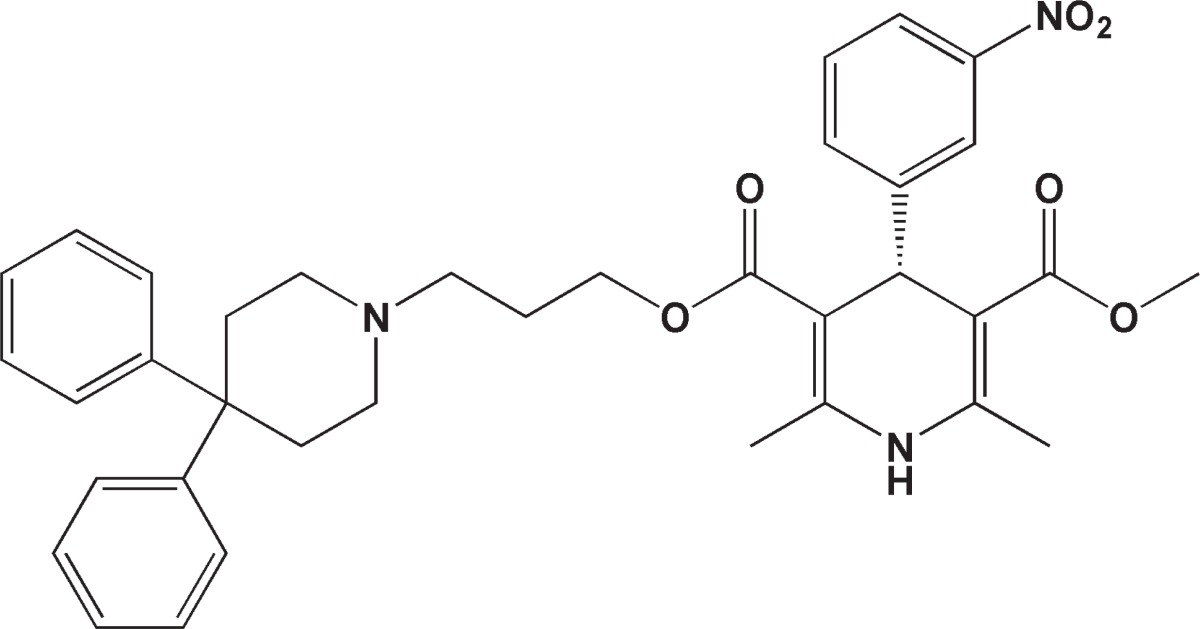

**Table 2:** Chemical structures of the 2,6-dimethyl-3,5-bis-*N*-(aryl/heteroaryl) carbamoyl-4-aryl-1,4-dihydropyridine derivatives (*VIIa*-*i*

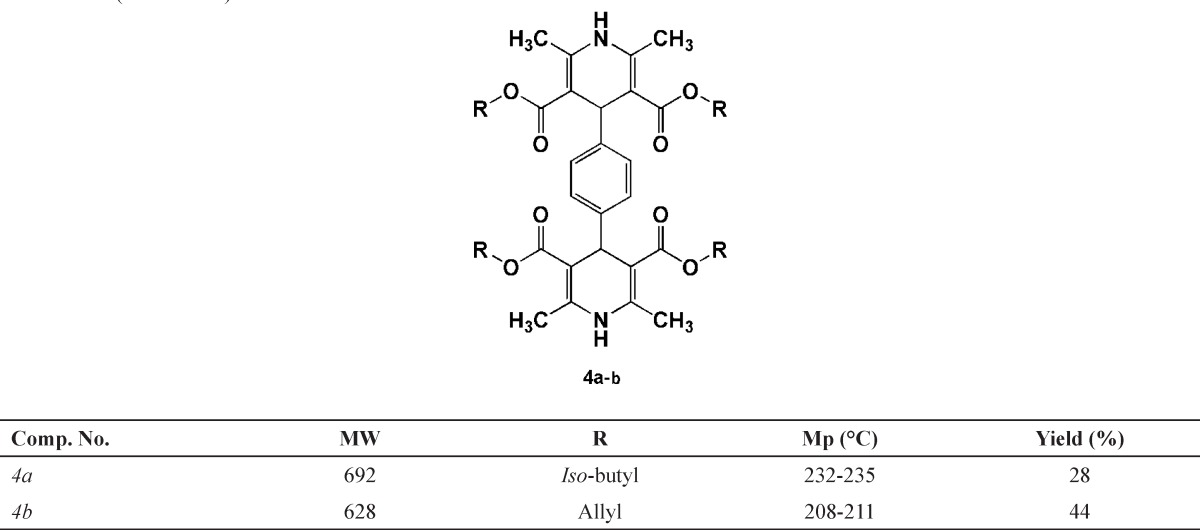

**Table 3:** Cytotoxic activity of 2,6-dimethyl-3,5-bis-*N*-(aryl/heteroaryl) carbamoyl-4-aryl-1,4-dihydropyridines assessed by the MTT reduction assay

**Compound No.**	**IC**_50 _ **(µM) **		
	**MCF-7**	**LS180**	**MOLT-4**
*IVa*	NA	NA	NA
*IVb*	NA	NA	NA
*VIIa*	28.8 ± 2.8	29.7 ± 4.7	17.4 ± 2.0
*VIIb*	52.4 ± 5.9	90.6 ± 7.3	55.9 ± 7.9
*VIIc*	56.1 ± 11.5	63.0 ± 5.1	32.8 ± 0.7
*VIId*	28.5 ± 3.5	47.0 ± 18.4	18.7 ± 1.2
*VIIe*	36.3 ± 3.1	71.4 ± 4.8	36.0 ± 0.7
*VIIf*	NA	NA	NA
*VIIg*	NA	NA	NA
*VIIh*	NA	NA	NA
*VIIi*	NA	NA	NA
Cisplatin	14.8 ± 8.1	5.0 ± 2.0	3.9 ± 0.3

**Scheme 2:**
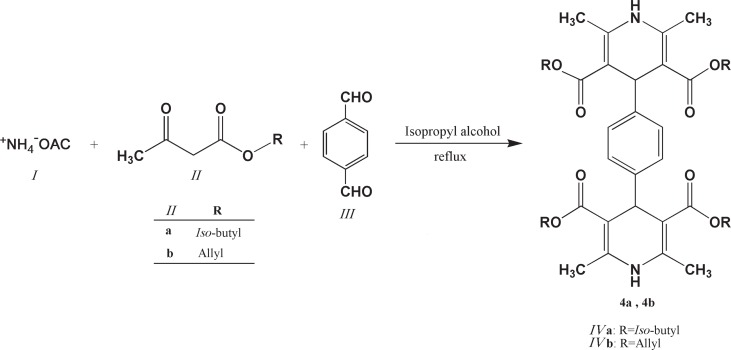
General synthetic route to 1,4-bis(2,6-dimethyl-3,5-dimethoxylcarbonyl-1,4-dihydropyridine-4-yl) benzenes (**IVa** and **IVb**

**Scheme 3.**
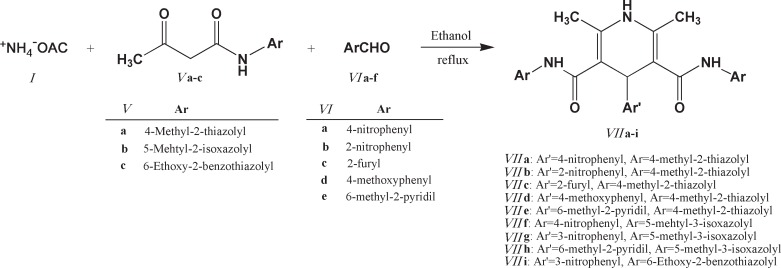
General synthetic route to 2,6-dimethyl-3,5-bis-*N*-(aryl/heteroaryl) carbamoyl-4-aryl-1,4-dihydropyridine (**VIIa-i**

## Results and Discussion

*Chemistry*

The structures of all synthesized 1,4-bis(2,6-dimethyl-3,5-dialkyloxylcarbonyl-1,4-dihydropyridine-4-yl) benzene and 2,6-dimethyl-3,5-bis-*N*-(aryl/heteroaryl) carbamoyl-4-aryl-1,4-dihydropyridines were confirmed by spectroscopic and elemental analysis methods. Schematic representation of the synthesis of compounds 4a, 4b and 7a-i are depicted in Schemes 2 and 3. 

*Cytotoxic activity*

Synthesized1,4-bis(2,6-dimethyl-3,5-dialkyloxylcarbonyl-1,4-dihydropyridine-4-yl) benzene and 2,6-dimethyl-3,5-bis-*N*-(aryl/heteroaryl)carbamoyl-4-aryl-1,4-dihydropyridine derivatives were assayed for their cytotoxicity against three human cancer cell lines (MCF-7, LS180, and MOLT-4) in terms of their IC_50_ values (Table 3.). It should be noted that none of the evaluated compounds showed equal or superior activity compared to cisplatin.

Considering the data in Table 3, the following structure activity relationships (SAR) may be envisaged:

DHP compounds bearing 3,5-bis-*N*-(4-methyl-2-thiazolyl) substituents (7a-e) showed superior cytotoxic effect compared to 3,5-bis-*N*-(5-methyl-3-isoxazolyl) substituted DHPs (7f-g). Higher activity of thiazole containing DHPs might be attributed to the role of sulfur atoms in enhancing the lipophilicity of the molecule and thus enhancing the penetration through cell membranes.

3,5-bis-*N*-(6-Ethoxy-2-benzothiazolyl) substituted DHP molecule (7i) did not show cytotoxic activity against MCF-7, LS180 and MOLT-4 cancer cell lines. This activity loss might be attributed to the possible steric hindrance of this derivative at the target site.

1,4-bis(2,6-dimethyl-3,5-dialkyloxylcarbonyl-1,4-dihydropyridine-4-yl) benzenes (4a and 4b) had no cytotoxic activity against any of the tested cells. One possible explanation to this is their bulky chemical structure leading to steric clash in their site of action.

Similar trend of activity could be observed for derivatives 7a-i against the LS180 and MOLT-4 cells. Due to the structural similarities of these compounds, observed order of activity could be related to their different substitution pattern on C4 of DHP ring (4-nitrophenyl > 4-methoxyphenyl > 2-furyl > 6-methyl-2-pyridil > 2-nitrophenyl). It appeared that DHP derivatives possessing H-acceptor/donor groups on their *para *position of C4-substituted aromatic ring showed superior activity against LS180 and MOLT-4 cells (Table 3.).

The presence of H-acceptor/donor groups on the *para *position of C4-substituted aromatic ring might be more determinant in MOLT-4 cells (7a; IC_50_=17.4 µM and 7d; IC_50_=18.7 µM).

## Conclusion

2,6-Dimethyl-3,5-bis-*N*-(aryl/heteroaryl) carbamoyl-4-aryl-1,4-dihydropyridine analogues showed weak to relatively good cytotoxic activity against MCF-7, LS180 and MOLT-4 human cancer lines, while a superior potency was observed in the case of MOLT-4 cells (7a: IC_50 _= 17.4±2.0 µM). It was revealed that 1,4-bis(2,6-dimethyl-3,5-dialkyloxylcarbonyl-1,4 dihydropyridine-4-yl) benzenes (4a and 4b) exhibited no cytotoxic effect on tested cancer cell lines possibly due to their bulky scaffold and hence steric hindrance in their site of action. The outcomes of this study may provide helpful information to guide the rational design and synthesis of more potent cytotoxic molecules on the basis of 1,4-DHP scaffold. 

## References

[1] Seffrin JR, Hill D, Burkart W, Magrath I, Badwe RA, Ngoma T, Mohar A, Grey N (2009). It is time to include cancer and other noncommunicable diseases in the millennium development goals. CA Cancer J. Clin..

[2] Thun MJ, DeLancey JO, Center MM, Jemal A, Ward EM (2010). The global burden of cancer: priorities for prevention. Carcinogenesis.

[3] Chabner BA, Roberts TG (2005). Chemotherapy and the war on cancer. Nat. Rev. Cancer.

[4] Davood A, Mansouri N, Dehpour AR, Shafaroudi H, Alipour E, Shafiee A (2006). Design, synthesis, and calcium channel antagonist activity of new 1,4-dihydropyridines containing 4-(5)-chloro-2-ethyl-5-(4)-imidazolyl substituent. Arch. Pharm. Chem. Life Sci..

[5] Shafiee A, Rastkary N, Jorjani M, Shafaghi B (2002). Synthesis and antihypertensive activities of new 1,4-dihydropyridine containing nitroimidazolyl substituent with a nitrooxy group at the 3- ester position. Arch. Pharm..

[6] Ogawa AK, Willoughby CA, Bergeron R, Ellsworth KP, Geissler WM, Myers RW, Yao J, Harris G, Chapman KT (2003). Glucose-lowering in a db/db mouse model by dihydropyridine diacid glycogen phosphorylase inhibitors. Bioorg. Med. Chem. Lett..

[7] Iman M, Davood A, Dehqani G, Lotfinia M, Sardari S, Azerang P, Amini M (2015). Design, synthesis and evaluation of Antitubercular activity of novel Dihydropyridine containing imidazolyl substituent. Iran. J. Pharm. Res..

[8] Kumar RS, Idhayadhulla A, Jamal Abdul Nassera A, Selvin J (2011). Synthesis and anticoagulant activity of a new series of 1, 4-dihydropyridine derivatives. Eur. J. Med. Chem..

[9] Kumar A, Maurya RA, Sharma S, Kumar M, Bhatia G (2010). Synthesis and biological evaluation of N-aryl-1, 4-dihydropyridines as novel antidyslipidemic and antioxidant agents. Eur. J. Med. Chem..

[10] Leon R, de los Rı´os C, Marco-Contelles J, Lo´pez MG, Garcı´a AG, Villarroya M (2008). Synthesis of 6-amino-1, 4-dihydropyridines that prevent calcium overload and neuronal death. Eur. J. Med. Chem..

[11] Kawase M, Shah A, Gaveriya H, Motohashi N, Sakagami H, Varga A, Molnar J (2002). 3,5-Dibenzoyl-1,4-dihydropyridines: synthesis and MDR reversal in tumor cells. Bioorg. Med. Chem..

[12] Firuzi O, Javidnia K, Mansourabadi E, Saso L, Mehdipour AR, Miri R (2013). Reversal of multidrug resistance in cancer cells by novel asymmetrical 1,4-dihydropyridines. Arch. Pharm. Res..

[13] Bazargan L, Fouladdel S, Shafiee A, Amini M, Ghaffari SM, Azizi E (2008). Evaluation of anticancer effects of newly synthesized dihydropyridine derivatives in comparison to verapamil and doxorubicin on T47D parental and resistant cell lines in vitro. Cell Biol. Toxicol..

[14] Lee YS, Sayeed MM, Wurster RD (1994). Inhibition of cell growth and intracellular Ca2+ mobilization in human brain tumor cells by Ca2+ channel antagonists. Mol. Chem. Neuropathol..

[15] Yoshida J, Ishibashi T, Nishio M (2003). Antiproliferative effect of Ca2+ channel blockers on human epidermoid carcinoma A431 cells. Eur. J. Pharmacol..

[16] Sirisha K, Achaiah G, Reddy VM (2010). Facile synthesis and antibacterial, antitubercular, and anticancer activities of novel 1,4-dihydropyridines. Arch. Pharm. Chem. Life Sci..

[17] Engi H, Sakagami H, Kawase M, Parecha A, Manvar D, Kothari H, Adlakha P, Shan A, Motohashi NIO, Molnar J (2006). Tumour-specific cytotoxicity and MDR-reversal activity of dihydropyridines. In Vivo.

[18] Foroughinia F, Javidnia K, Amirghofran Z, Mehdipour AR, Mir R (2008). Design and synthesis of new symmetrical derivatives of dihydropyridine containing a pyridyl group on the 3, 5-positions and evaluation of their cytotoxic and multidrug resistance reversal activity. J. Pharm. Pharmacol..

[19] Abbas HAS, El Sayed WA, Fathy NM (2010). Synthesis and antitumor activity of new dihydropyridine thioglycosides and their corresponding dehydrogenated forms. Eur. J. Med. Chem..

[20] Hahn KA, Legendre AM, Schuller HM (1997). Amputation and dexniguldipine as treatment for canine appendicular osteosarcoma. J. Cancer Res. Clin..

[21] Zhu LJ, Zhuang CL, Lei N, Sheng CQ, Guo W, Miao ZY, Liu WF, Yao JZ, Zhang WN (2011). Synthesis and pharmacological evaluation of novel homocamptothecin-dihydropyridine derivative conjugates as potent topoisomerase I inhibitors. Aust. J. Chem..

[22] Miri R, Javidnia K, Hemmateenejad B, Azarpira A, Amirghofran Z (2004). Synthesis, cytotoxicity, QSAR, and intercalation study of new diindenopyridine derivatives. Bioorg. Med. Chem..

[23] Miri R, Motamedi R, Rezaei MR, Firuzi O, Javidnia A, Shafiee A (2010). Design, synthesis and evaluation of cytotoxicity of novel chromeno [4,3-b] quinoline derivatives. Arch. Pharm..

[24] Sarkarzadeh H, Miri R, Firuzi O, Amini M, Razzaghi-Asl N, Edraki N, Shafiee A (2013). Synthesis and antiproliferative activity evaluation of imidazole-based indeno[1,2-b]quinoline-9,11-dione derivatives. Arch. Pharm. Res..

[25] Clemens RJ, Hyatt JA (1985). Acetoacetylation with 2,2,6-trimethyl-4H-1,3-dioxin-4-one: a convenient alternative to diketene. J. Org. Chem..

[26] Razzaghi-Asl N, Firuzi O, Hemmateenejad B, Javidnia K, Edraki N, Miri R (2013). Design and synthesis of Novel 3,5-bis-N-(aryl/heteroaryl) carbamoyl-4-aryl-1,4-dihydropyridines as small molecule BACE-1 inhibitors. Bioorg. Med. Chem..

[27] Mohammadhosseini N, Pordeli M, Safavi M, Firoozpour L, Amin F, Ardestani SK, Edraki N, Shafiee A, Foroumadi A (2015). Novel n-2-(Furyl)-2-(chlorobenzyloxyimino) ethyl piperazinyl quinolones: Synthesis, cytotoxic evaluation and structure-activity relationship. Iran. J. Pharm. Res..

